# A new model for predicting boiling points of alkanes

**DOI:** 10.1038/s41598-021-03541-z

**Published:** 2021-12-20

**Authors:** Simon Mukwembi, Farai Nyabadza

**Affiliations:** 1 School of Mathematics, University of the Witwatersrand, Johannesburg, South Africa; 2 Department of Mathematics and Applied Mathematics, University of Johannesburg, Johannesburg, South Africa

**Keywords:** Biochemistry, Chemistry, Mathematics and computing

## Abstract

A general perception among researchers is that boiling points, which is a key property in the optimization of lubricant performance, are difficult to predict successfully using a single-parameter model. In this contribution, we propose a new graph parameter which we call, for lack of better terminology, the conduction of a graph. We exploit the conduction of a graph to develop a single-parameter model for predicting the boiling point of any given alkane. The model was used to predict the boiling points for three sets of test data and predicted with a coefficient of determination, $R^2=0.7516,~0.7898$ and 0.6488, respectively. The accuracy of our model compares favourably to the accuracy of experimental data in the literature. Our results have significant implications on the estimation of boiling points of chemical compounds in the absence of experimental data.

## Introduction

A *graph* $G=(V,E)$ is a mathematical object which consists of a finite set *V* of elements called *vertices*, together with a set *E*, of 2-element subsets of *V*, called the *edges* of *G*. As early as 1875, Cayley (see for instance^1^), in his quest to enumerate chemical molecules called alkanes, he made an observation that molecules can be modelled by graphs where atoms are represented by vertices and two vertices are joined by an edge if the corresponding atoms are linked by bonds. This graph model became widely known as the *molecular graph*. An interesting numeric value attached to each molecule is the boiling point, i.e., the temperature at which a substance has a vapour pressure of 760 mmHg^2^. Whilst the boiling point is a key property in classifying molecules, such as alkanes which dominate mixtures of lubricants in industry, there is generally lack of experimental boiling point data (see, for instance^1^ and references cited therein). Nevertheless, although expected to be accurate—experimental boiling points are sometimes inaccurate due to the presence of impurities, and as a result, have wide discrepancies especially for higher boiling points. This has the propensity to lead to wider and elevated boiling point ranges. For instance, the boiling point of cyclooctane (an alkane) is reported to range from $120.3$ to $156.8\;^\circ$C, and that of methylcycloheptane ranges from $113$ to $136.8\;^\circ$C^2^. The lack of data, and the inaccuracies where data is available, have necessitated the development of boiling point models which can be used to estimate boiling points of chemical compounds for which no boiling point data is available or where inaccuracies exist. A natural foundation on which these models are built are graph parameters^3,4^.

The Wiener index of a graph, introduced by Harold Wiener in 1947^5^, was the first graph parameter to be used in chemistry. In particular, it was used to predict boiling points of alkanes. Ever since its introduction, a lot of effort was invested in research that gave rise to the development of extensions of the Wiener index^3^, and of other indices such as the Hosoya index^3^, the Gutman and the Schultz indices^6^, and a legion of other distance-based topological indices (see, for example^7^), which are used for the prediction of various properties of molecules.

For alkanes, single variable models that are available are either weak^3^ or they consider only a special class of the molecules^1^. Burch, Wakefield and Whitehead^1^ successfully developed a single variable model to calculate boiling points of special families of alkanes and developed multivariate models that can predict boiling points of all alkanes up to and including those of order 12. Several other models, see for instance^8–10^, abound in literature with the most recent one being due to Sandak and Conduit^11^ who trained artificial neural networks to predict the physical properties of linear, single branched, and double branched alkanes. Clearly, as Dearden^2^, pointed out several years ago, modeling a property is easier when one is dealing with a single chemical class. However, from the point of view of an engineer concerned with a wide range of compounds, the methods of greater interest are those that can adequately model the behavior of varied data sets.

In this paper, we are concerned with relating two mathematical objects, associated with a chemical molecule, namely the molecular graph and the numeric value attached to the boiling point of the molecule. We focus on molecular graphs for all alkanes and introduce a new graph parameter which, we define as the ‘conduction’. We use this new parameter, the conduction, to develop a single-variable model that predicts the boiling point of any given alkane. We use^1^ as the source for the experimental data of boiling points.

This paper is organised as follows: in the next section, we introduce the new graph parameter, the conduction of a graph, and illustrate how it is computed by determining the conduction of four special classes of graphs representing some important series of alkanes. The model is developed in the “Model development”. The main results are presented in “Main results” in which the model is tested. This will be followed by the discussion and the conclusion is “Discussion and conclusion”.

## The graph parameter: conduction

Consider a connected graph $G=(V,E)$ of order *n*. The *distance* $d_G(u,v)$ between vertices *u* and *v* in *G* is defined as the length of a shortest path joining *u* and *v* in *G*. For vertex *v* denote by $T_v$, the breadth-first search tree of *G* based at *v*, and let $S_v$ be the set of end vertices of $T_v$. We select $T_v$ to be the tree that minimizes $\sum _{x\in S_v}d(v,x)$ amongst all breadth-first search trees of *G* based at *v*. To contrive the conduction parameter, we envisage heat flowing from one atom of a chemical compound to the other atoms. We assume that in the heat conduction process between atoms (depicted by vertices of a graph), heat is transferred from vertex *v* outwardly to all other vertices in the graph through contact via the edges of the tree $T_v$ until it reaches the end vertices in $T_v$. The speed of transfer, conceivably, is governed by the breadth of $T_v$ which can be approximated by the square of the degree, $\mathrm{deg}~v$, of vertex *v*. As a result, in the heat conduction process, let the *score* *s*(*v*) of vertex *v* be the quantity

$$\begin{aligned} s(v)=\mathrm{deg}^2v[\sum _{x\in S_v}d(v,x)]. \end{aligned}$$

Mathematically, the *conduction* of a graph *G*, *c*(*G*),  is defined as

$$\begin{aligned} c(G)=\frac{1}{n}\sum _{v\in V(G)}s(v). \end{aligned}$$

**Figure 1:**
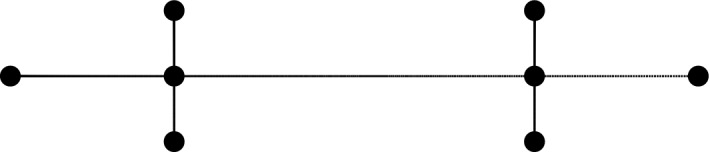
Molecular graph for the alkane, 2,2,3,3-tetramethylbutane.

For instance, the graph in Fig. 1 has conduction $\frac{366}{8}$.

We use four special classes of graphs. The first is the path, $P_n$, of order *n*. The *broom graph*, $B_{n,q}$, is a graph of order *n* obtained by taking the path $P_{n-q}$ and attaching *q* end vertices to one end of $P_{n-q}$. The second class of graphs we will consider is $B_{n,2}$ while the third class is $B_{n,3}$. These classes of graphs, namely $P_n=B_{n,1}$, $B_{n,2}$ and $B_{n,3}$, represent the class of normal-alkanes, the 2-methyl, and the 2,2-dimethyl series of alkanes, respectively. The fourth class is that of graphs $E_n$ of order *n* where $E_n$ has fairly large conduction among all graphs of order *n*, and $E_n$ consists of a path *P*, and having as many vertices of degree 4 on *P* as possible. For instance, $E_8$ is given in Fig. 1. Table 1 shows some of the graphs, $E_n$. In Tables 1, 2, 3, 4, 5, 6, 7, 8 and 9, $\mathbf{c}$ is the conduction.

**Table 1:** Some $E_n$ graphs.

Number	Alkane	*c*	Boiling point in $^\circ$C
$E_5$	2,2-Dimethylpropane	17.6	9.5
$E_6$	2,3-Dimethylbutane	23.33	58.1
$E_7$	2,2,3-Trimethylbutane	33.71	81
$E_8$	2,2,3,3-Tetramethylbutane	45.75	106.5
$E_9$	2,3,3,4-Tetramethylpentane	52	141.5
$E_{10}$	2,2,3,3,4-Pentamethylpentane	66.3	161.1

A simple calculation establishes the following formulae.

### Proposition 2.1

*Let* $$n\ge 4$$.* Then *

1. $$c(P_n)=4n-10+\frac{6}{n}$$,
2. $$c(B_{n,2})=6n-21+\frac{27}{n}$$,
3. $$c(B_{n,3})=8n-38+\frac{78}{n}.$$

Using data in Table 1, we can approximate the conduction of $E_n$ by an equation of the form

1 $$\begin{aligned} c(E_n)=\alpha n^2+\beta n+\gamma +\lambda \frac{1}{n}, \end{aligned}$$

where $\alpha ,\beta ,\gamma$ and $\lambda$ are constants.

Fitting data in Table 1 to Eq. (1), we get the following equation with a *coefficient of determination*, CoD, $R^2=0.9924.$ We discuss in detail the concept of the CoD is “Model quality assessment”.

### Proposition 2.2

*Let* $$n\ge 5$$.* Then*

$$\begin{aligned} c(E_n)\approx \frac{4}{9}n^2+3.44857532n-14.95083692+\frac{18.25213877}{n}. \end{aligned}$$

## Model development

### Model quality assessment

In the development process of our model, we adopt the folkore method, goodness of fit, of determining the quality of the model. After performing a fitting process, it is important to determine the goodness of the fit. While there are many methods of determining the goodness of a fit, in this paper we evaluate the residuals and make a plot of the residuals. The evaluation of the residuals is important in determining the goodness of fit of our model to data. We use the CoD, that is used to explain the variability between our model output and the data. This coefficient is commonly known as R-squared, ($R^2$). The formula of coefficient of determination is given by:

$$\begin{aligned} R^2 = 1 -\frac{\hbox {RSS}}{\hbox {TSS}}, \end{aligned}$$

where, $$R^2 = \mathrm{Coefficient~of~ Determination}$$, $${\mathrm{RSS}} = \mathrm{Residuals~ sum~ of~ squares}$$ and $$\mathrm{TSS} = \mathrm{Total~ sum~ of~squares}.$$

A CoD value of 1 indicates a perfect fit, and thus the model can be deemed very reliable for any future forecasts, while a value of 0 indicates that the model does not accurately model the data. Some researchers have argued that it is better to look at adjusted R-squared rather than the R-squared, but for the work presented in this paper, it suffices to use the non-adjusted R-squared.

### Problem statement

Let $A_n$ be an alkane with *n* carbon atoms. Our problem is to estimate the boiling point of the alkane, $A_n$. We will represent this by a graph, $G_n$, of order *n*. We will often use $A_n$ and $G_n$ interchangeably. We will thus denote the boiling point of $A_n$ by $b(A_n)$ or $b(G_n).$

### Model when sufficient data is available

We first consider a situation in which there is sufficient data on the boiling points of a group of alkanes. The general observation is that the conduction values of alkanes of order *n* have a global linear relationship with the experimental boiling points and there are localised oscillations about the regression line where the diameter of the oscillations decreases as conduction increases. In general, as a first approximation, to find $b(A_n)$, we propose a model of the form:

2 $$\begin{aligned} b(A_n)=\alpha c(G_n)+\beta , \end{aligned}$$

where $c(G_n)$ is the conduction of graph $G_n$ and $\alpha$ and $\beta$ are constants to be determined by fitting the model to boiling points data of alkanes of order *n*. To improve the fit and accuracy, we propose a combination of linear fit with logarithmic and trigonometric functions. The logarithmic and trigonometric components are incorporated to capture the oscillatory tendencies. We thus propose a function of the form,

3 $$\begin{aligned} b(A_n)=\alpha c(G_n)+\beta + \alpha _1\log _{\alpha _2}[\alpha _3c(G_n)+\alpha _4]\sin [\alpha _5 c(G_n)+\alpha _6], \end{aligned}$$

where $\alpha , ~\beta$, $\alpha _i$, $i=1,2,\ldots ,6$, are constants to be determined by fitting the model to data of alkanes of order *n*.

To illustrate the application of (3), we consider the data set depicted in Table 2 of all alkanes of order 6.

**Table 2:** Data for 5 alkanes with 6 carbon atoms.

Number	Alkane	*c*	Boiling point in $^\circ$C
1	Hexane	15	68.8
2	3-Methylpentane	18.83	63.3
3	2-Methylpentane	19.5	60.9
4	2,2-Dimethylbutane	23	49.8
5	2,3-Dimethylbutane	23.33	58.1

**Figure 2:**
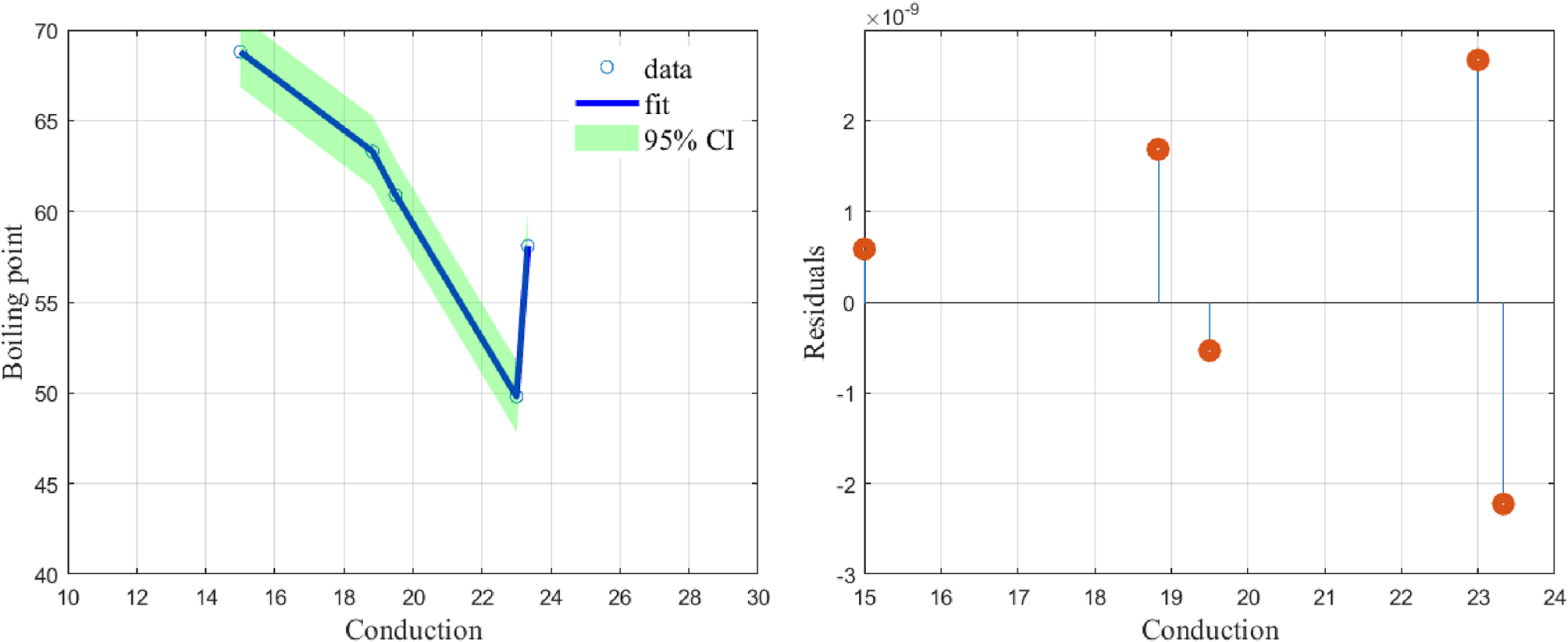
shows the model fit with a 95% confidence interval and the residuals.

While a linear model, (2), fits the data with $(\alpha ,\beta )=(-1.8245, 96.5461)$ and a CoD value of $R^2=0.7903$ our model, (3), fits the data with

$$\begin{aligned} (\alpha ,\beta ,\alpha _i)= (2.7173,-0.2030,24.5237,3.5331,0.4679,-6.7879,-0.9061,18.1828) \end{aligned}$$

and a CoD value of $R^2=1$. The graphs of the fit using the least squares (lsq) fitting method and the residuals are given in Fig. 2. It is clear that the proposed function (3) fits well to the data in Table 2 with very low values of the residuals.

### Model with insufficient data

Our model to estimate the boiling point of an alkane $A_n$ of order *n* depends on the availability of data for alkanes of order *n*. In the absence of such data, our initial step will be to develop the data. Once we develop the data, we can then use (2) [or (3), for more accuracy] to find the equation that estimates the boiling point to a reasonable degree of accuracy (namely, coefficient of determination values of $R^2 = 0,7516, 0,7898$ and 0, 6488 for three test data sets, respectively). We cover this below.

#### Generating data

We need to generate data for some alkanes of order *n* as more data yields better accuracy during the fitting process. The graphs $P_n$, $B_{n,2}$, $B_{n,3}$ and $E_n$ are all alkanes of order *n* we estimate their boiling points in turn below.

#### Boiling point for $P_n$

Comparing the boiling points of normal alkanes with their conduction values, we see that growth of boiling points of the normal-alkanes is linear in conduction values but with an additional logarithmic increase added to it. We, thus, propose the model

$$\begin{aligned} b(P_n)=\alpha c(P_n)+\beta + \alpha _1\log _{\alpha _2}[\alpha _3c(P_n)+\alpha _4], \end{aligned}$$

where $\alpha , ~\beta$, $\alpha _i$, $i=1,2,3,4$, are constants to be determined by fitting the model to data of normal-alkanes. From Proposition 2.1, this reduces to

4 $$\begin{aligned} b(P_n)=\alpha \left( 4n-10+\frac{6}{n}\right) +\beta + \alpha _1\log _{\alpha _2}\left[ \alpha _3\left( 4n-10+\frac{6}{n}\right) +\alpha _4\right] . \end{aligned}$$

Fitting (4) to the data of the first few normal-alkanes given in Table 3, we obtain the values of the constants as

$$\begin{aligned} (\alpha ,\beta ,\alpha _1,\alpha _2,\alpha _3,\alpha _4)= (1.5911,20.9101,41.6488,1.2703,0.0429,0.5061) \end{aligned}$$

with a CoD value of $R^2=0.9999$.

**Table 3:** Data for 11, normal-alkanes.

Number	Alkane	*c*	Boiling point in $^\circ$C
1	Butane	7.5	0
2	Pentane	11.2	36.1
3	Hexane	15	68.8
4	Heptane	18.85714	98.38
5	Octane	22.75	125.6
6	Nonane	26.66667	150.7
7	Decane	30.6	174.1
8	Undecane	34.54545	195
9	Dodecane	38.5	216
10	Tridecane	42.46154	234
11	Tetradecane	46.42857	255

**Figure 3:**
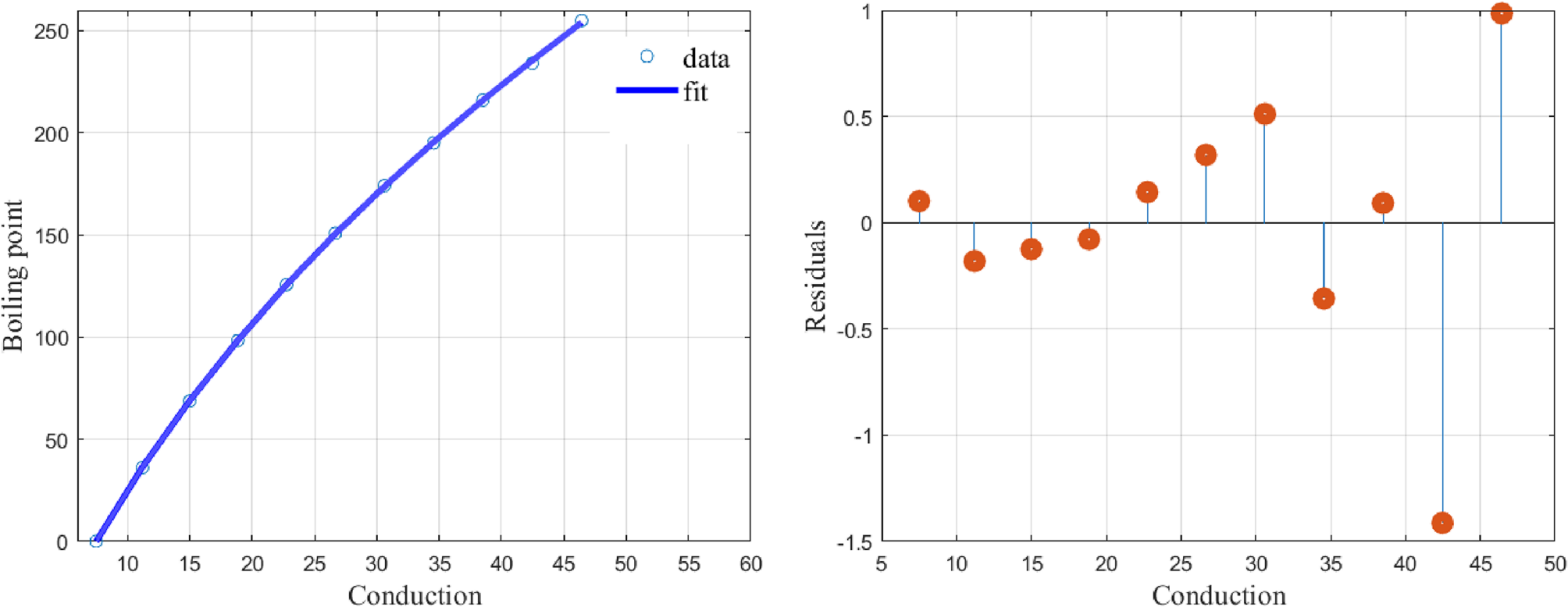
Shows the model fit and the residuals.

The graph and the plot of residuals are given in Fig. 3.

The proposed function (4) fits well to the data in Table 3 and the residual values are also very low.

#### Boiling point for $B_{n,2}$

As in the case of normal-alkanes, we propose the model

$$\begin{aligned} b(B_{n,2})=\alpha c(B_{n,2})+\beta + \alpha _1\log _{\alpha _2}[\alpha _3c(B_{n,2})+\alpha _4], \end{aligned}$$

where $\alpha , ~\beta$, $\alpha _i$, $i=1,2,3,4$, are constants to be determined by fitting the model to data of 2-methyl series of alkanes. From Proposition 2.1, this reduces to

5 $$\begin{aligned} b(B_{n,2})=\alpha \left( 6n-21+\frac{27}{n}\right) +\beta + \alpha _1\log _{\alpha _2}\left[ \alpha _3\left( 6n-21+\frac{27}{n}\right) +\alpha _4\right] . \end{aligned}$$

Fitting (5) to the data of the first few 2-methyl series given in Table 4, we obtain the values of the constants as

$$\begin{aligned} (\alpha ,\beta ,\alpha _1,\alpha _2,\alpha _3,\alpha _4)= (1.2125,23.5857,40.7654,1.3216,0.03559,0.3998) \end{aligned}$$

with a CoD value of $R^2=0.9996$.

**Table 4:** Data for 8, 2-methyl series.

Number	Alkane	*c*	Boiling point in $^\circ$C
1	2-Methylbutane	14.5	27.4
2	2-Methylpentane	19.5	60.9
3	2-Methylhexane	24.85714	90.1
4	2-Methylheptane	30.375	117.6
5	2-Methyloctane	36	143
6	2-Methylnonane	41.7	166.9
7	*2-Methyldecane*	47.45455	189.3
8	*2-Methylundecane*	53.25	208.9
9	2-Methyldodecane	59.07692	229.5
10	2-Methyltridecane	64.92857	247.9

**Figure 4:**
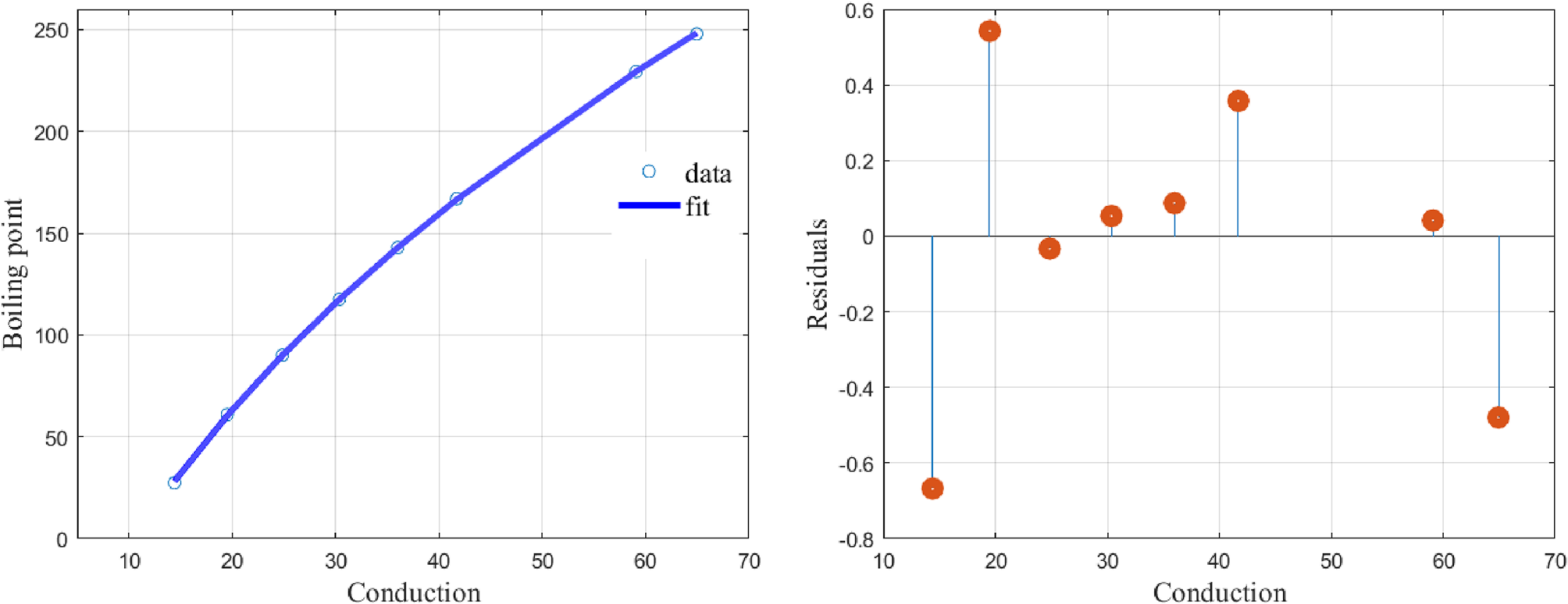
Shows the model fit and the residuals.

The graph and the plot of residuals are given in Fig. 4.

**Figure 5:**
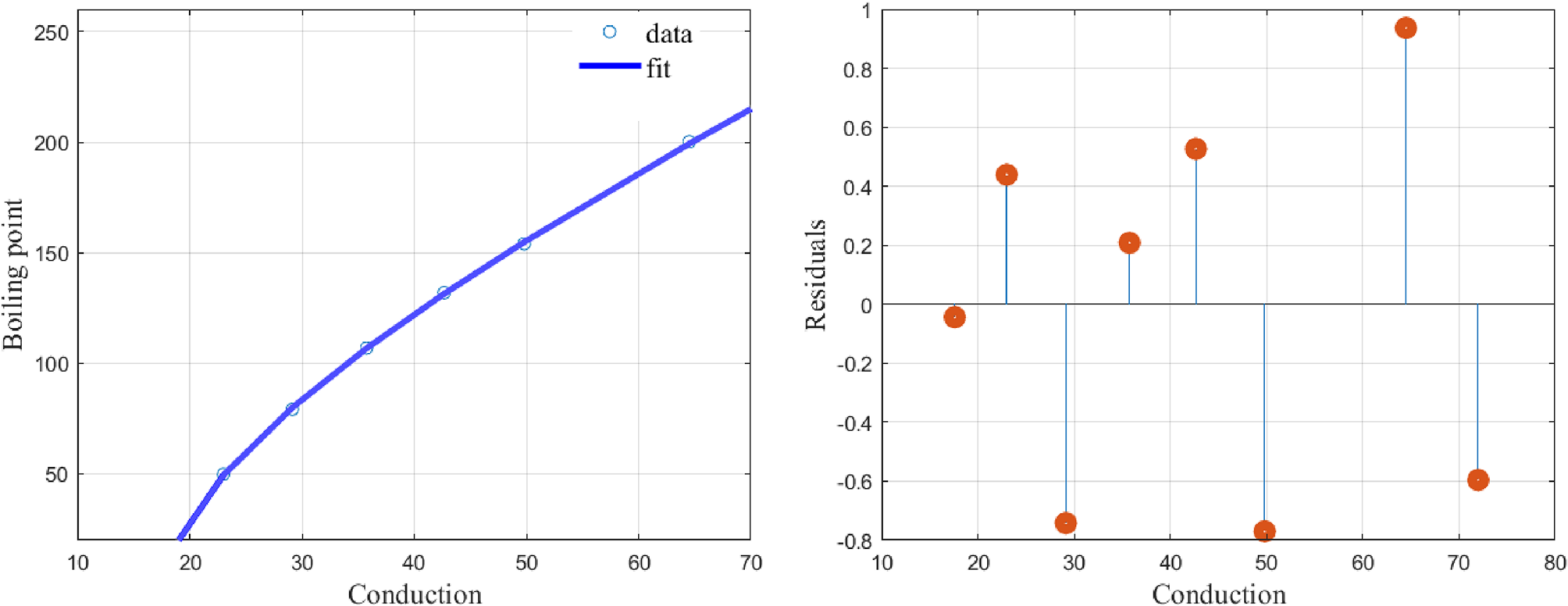
Shows the model fit and the residuals.

**Figure 6:**
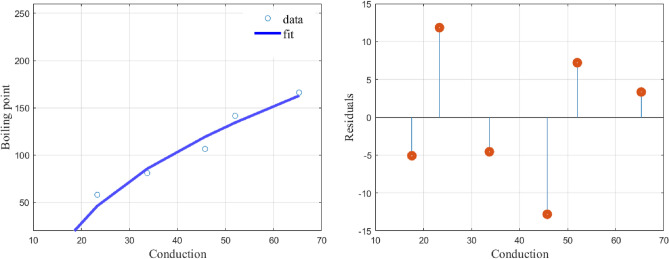
Shows the model fit and the residuals.

The function (5) produces a perfect fit to the data in Table 4 and the resulting residual values are very low. similar observations are made for Figs. 5 and 6.

#### Boiling point for $B_{n,3}$

As in the case of normal-alkanes, we propose the model

$$\begin{aligned} b(B_{n,3})=\alpha c(B_{n,3})+\beta + \alpha _1\log _{\alpha _2}[\alpha _3c(B_{n,3})+\alpha _4], \end{aligned}$$

where $\alpha , ~\beta$, $\alpha _i$, $i=1,2,3,4$, are constants to be determined by fitting the model to data of 2-dimethyl series of alkanes. From Proposition 2.1, this reduces to

6 $$\begin{aligned} b(B_{n,3})=\alpha \left( 8n-38+\frac{78}{n}\right) +\beta + \alpha _1\log _{\alpha _2}\left[ \alpha _3\left( 8n-38+\frac{78}{n}\right) +\alpha _4\right] . \end{aligned}$$

Fitting (6) to the data of the first few 2-dimethyl series given in Table 5, we obtain the values of the constants as

$$\begin{aligned} (\alpha ,\beta ,\alpha _1,\alpha _2,\alpha _3,\alpha _4)= (2.2207,15.9874,9.8662,1.3271,0.0613,-0.8074) \end{aligned}$$

with a CoD value of $R^2=0.9999$.

**Table 5:** Data for 8, 2,2-dimethyl series.

Number	Alkane	*c*	Boiling point in $^\circ$C
1	2,2-Dimethylpropane	17.6	9.5
2	2,2-Dimethylbutane	23	49.8
3	2,2-Dimethylpentane	29.14286	79.2
4	2,2-Dimethylhexane	35.75	106.9
5	2,2-Dimethylheptane	42.66667	131.9
6	2,2-Dimethyloctane	49.8	154
7	*2,2-Dimethylnonane*		
8	2,2-Dimethyldecane	64.5	200.12
9	2,2-Dimethylundecane	72	220
10	*2,2-Dimethyldodecane*		

The graph and the plot of residuals are given in Fig. 5.

#### Boiling point for $E_{n}$

As in the case of normal-alkanes, we propose the model

$$\begin{aligned} b(E_n)=\alpha c(E_{n})+\beta + \alpha _1\log _{\alpha _2}[\alpha _3c(E_{n})+\alpha _4], \end{aligned}$$

where $\alpha , ~\beta$, $\alpha _i$, $i=1,2,3,4$, are constants to be determined by fitting the model to data given in Table 1. From Proposition 2.2, this reduces to

7 $$\begin{aligned} b(E_{n})= & {} \alpha \left( \frac{4}{9}n^2+3.44857532n-14.95083692+\frac{18.25213877}{n}\right) +\beta \nonumber \\&+\alpha _1\log _{\alpha _2}\left[ \alpha _3\left( \frac{4}{9}n^2+3.44857532n-14.95083692+\frac{18.25213877}{n}\right) +\alpha _4\right] . \end{aligned}$$

Fitting (7) to the data given in Table 1, we obtain the values of the constants as

$$\begin{aligned} (\alpha ,\beta ,\alpha _1,\alpha _2,\alpha _3,\alpha _4)=(1.0404,-360.7375,4.2800,1.0802, 63.5399,-499.3487) \end{aligned}$$

with a CoD value of $R^2=0.9751$. The graph and the plot of residuals are given in Fig. 6.

## Main results

From the previous sections, we have now generated data for boiling points of some alkanes with *n* carbon atoms. Let

$$\begin{aligned}&x_1=4n-10+\frac{6}{n}, \\&x_2=6n-21+\frac{27}{n}, \\&x_3=8n-38+\frac{78}{n}, \end{aligned}$$

and

$$\begin{aligned} x_4=\frac{4}{9}n^2+3.44857532n-14.95083692+\frac{18.25213877}{n}. \end{aligned}$$

We present the data in Table 6.

**Table 6:** Data for 4 alkanes of order *n*.

Alkane	c	Boiling point in $^\circ$C
$P_n$	$x_1$	$y_1=\alpha \left( x_1\right) +\beta + \alpha _1\log _{\alpha _2}\left[ \alpha _3\left( x_1\right) +\alpha _4\right]$
		Where constants are as given in (4)
$B_{n,2}$	$x_2$	$y_2=\alpha \left( x_2\right) +\beta + \alpha _1\log _{\alpha _2}\left[ \alpha _3\left( x_2\right) +\alpha _4\right]$
		Where constants are as given in (5)
$B_{n,3}$	$x_3$	$y_3=\alpha \left( x_3\right) +\beta + \alpha _1\log _{\alpha _2}\left[ \alpha _3\left( x_3\right) +\alpha _4\right]$
		Where constants are as given in (6)
$E_{n}$	$x_4$	$y_4=\alpha \left( x_4\right) +\beta + \alpha _1\log _{\alpha _2}\left[ \alpha _3\left( x_4\right) +\alpha _4\right]$
		Where constants are as given in (7)

To find $b(A_n)$, we propose the lsq model:

8 $$\begin{aligned} b(A_n)=\lambda \cdot c(G_n)+\gamma , \end{aligned}$$

where

$$\begin{aligned} \lambda =\frac{x_1y_1+x_2y_2+x_3y_3+x_4y_4-\frac{1}{3}(x_1+x_2+x_3+x_4)(y_1+y_2+y_3+y_4)}{x_1^2+x_2^2+x_3^2+x_4^2-\frac{1}{4}(x_1+x_2+x_3+x_4)^2} \end{aligned}$$

and

$$\begin{aligned} \gamma= & {} \frac{1}{4}(y_1+y_2+y_3+y_4)-\frac{1}{4}(x_1+x_2+x_3+x_4)\lambda . \end{aligned}$$

### Model testing

We first test the predictive ability of our model, (8), on all alkanes of order 6. We present the results in Table 7. In Tables 7, 8 and 9, **Bp in** $^\circ$**C** means the experimental boiling points in degree Celsius, while **predicted** means the boiling points predicted by our model.

**Table 7:** All alkanes of order 6: Predictive ability of model (8).

Number	Alkane	c	Bp in $^\circ$C	Predicted
1	2-Methylpentane	19.5	60.9	59.8
2	3-Methylpentane	18.8	63.3	61.1
3	2,2-Dimethylbutane	23	49.8	53.1
4	2,3-Dimethylbutane	23.3	58.1	52.4
5	Hexane	15	68.8	68.5

**Figure 7:**
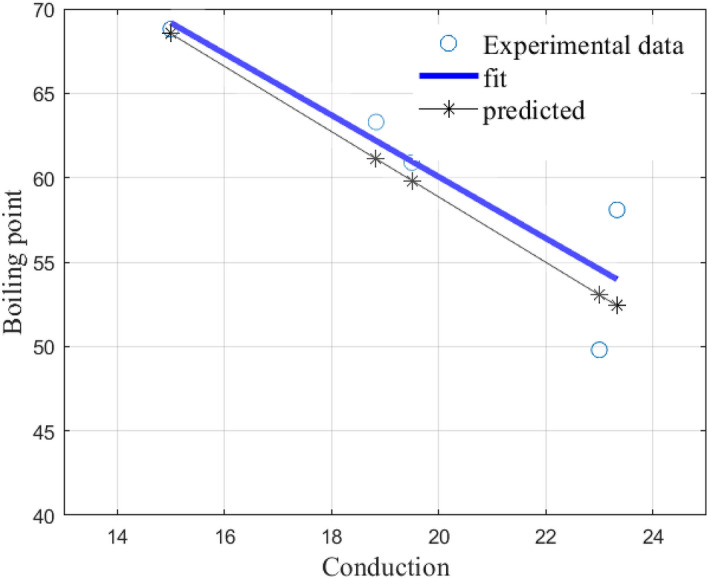
Shows a comparison of the linear least squares fit to experimental data and the linear model (8) for all alkanes of order 6.

The CoD value is $R^2=0.7516$ and the graphs are presented in Fig. 7 below.

Next we test the predictive ability of our model, (8), on all alkanes of order 7. We present the results in Table 8.

**Table 8:** All alkanes of order 6: Predictive ability of model (8).

Number	Alkane	c	Bp in $^\circ$C	Predicted
1	2,2,3-Trimethylbutane	33.71	80.9	80.7
2	3-Ethylpentane	23.14	93.5	92.1
3	3,3-Dimethylpentane	28	86.1	86.9
4	2,4-Dimethylpentane	30.86	80.6	83.8
5	2,3-Dimethylpentane	28.57	89.8	86.2
6	2,2-Dimethylpentane	29.14	79.2	85.6
7	2-Methylhexane	24.86	90.1	90.3

**Figure 8:**
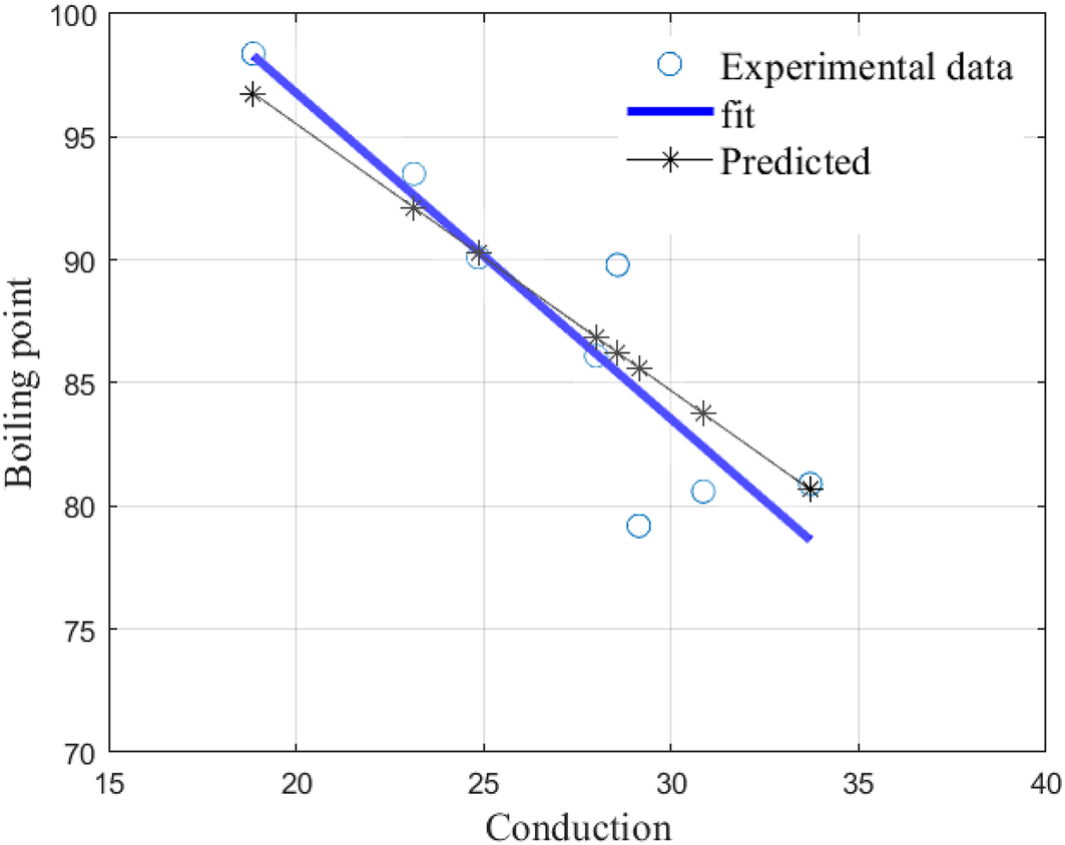
Shows a comparison of the linear least squares fit to experimental data and the linear model (8) for all alkanes of order 7.

The CoD value is $R^2=0.7898$ and the graphs are presented in Fig. 8 below.

We now test the predictive power of our model, (8), on alkanes of order 8. We present the results in Table 9.

**Table 9:** All alkanes of order 8: Predictive ability of model (8).

Number	Alkane	c	Bp in $^\circ$C	Predicted
1	2,2,3,3-Tetramethylbutane	45.75	106.5	106.7
2	2,2,3-Trimethylpentane	39.625	110	111.1
3	2,3,3-Trimethylpentane	39.125	114.7	111.4
4	2,2,4-Trimethylpentane	43.375	99.2	108.4
5	2,2-Dimethylhexane	35.75	106.9	113.8
6	3,3-Dimethylhexane	33.75	112	115.2
7	3-Ethyl-3-methylpentane	32.75	118.2	115.9
8	2,3,4-Trimethylpentane	40.625	113.4	110.4
9	2,3-Dimethy lhexane	34.5	115.6	114.7
10	3-Ethyl-2-methylpentane	33.5	115.6	115.4
11	3,4-Dimethylhexane	33.5	117.7	115.4
12	2,4-Dimethylhexane	36	109.4	113
13	2,5-Dimethylhexane	38.5	109	111.9
14	2-Methylheptane	30.375	117.6	117.6
15	3-Methylheptane	25.875	118	120.8
16	4-Methylheptane	28.375	117.7	119
17	3-Ethy lhexane	27.875	118.5	119.4
18	Octane	22.75	125.7	123

**Figure 9:**
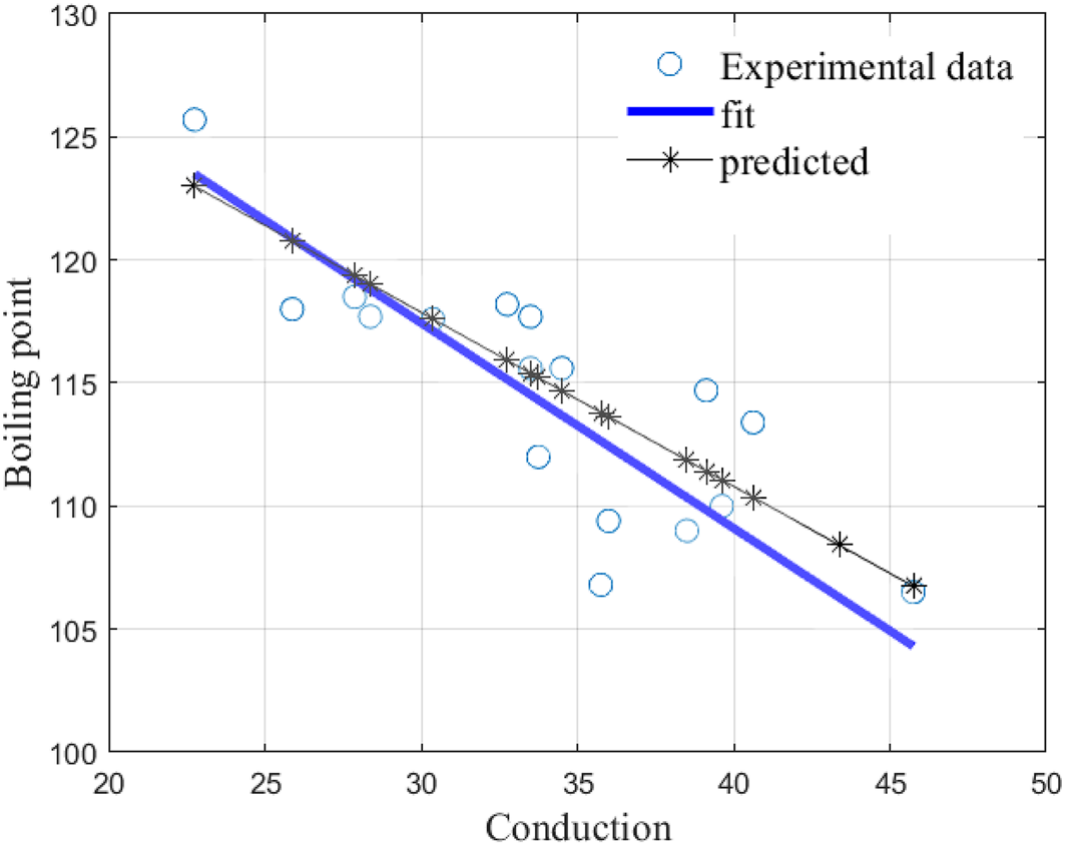
Shows a comparison of the linear least squares fit to experimental data and the linear model (8) for all alkanes of order 8.

The CoD value is $R^2=0.6488$ and the graphs are presented in Fig. 9 below.

## Discussion and conclusion

In this paper we provide a novel model for predicting the boiling points of alkanes. We consider alkanes of orders 6, 7 and 8 to test the usefulness of our model. We consider alkanes with *n* carbon atoms represented by a graph $G_n$ whose boiling points may be known experimentally. In particular, the methods presented here have the ability to develop the boiling points data using the conduction parameter, which we defined as the conduction of a graph. The nature of the boiling points data necessitated the use of a combination of trigonometric and logarithmic functions in coming up with models that fit to the data. The models are then used to predict the boiling points of alkanes whose number of carbon atoms are known.

The model presented in this article is without inculpability. First we fit linear models to experimental data that has very few number of data points in some instances. Second, some boiling points of certain compounds could not be ascertained from literature and last, the approximation of the numerical values of some parameters give rise to variability in the model predictions.

Despite these shortcomings, the model presents some strengths to literature. Firstly, our single-variable model puts to rest the general perception among researchers that boiling points are difficult to predict successfully using a single-parameter model^3,4^. Our key contribution has therefore been the development of a new parameter, the conduction of a graph, which could adequately capture the boiling points. In predicting boiling points, the conduction of a graph has proved to be more superior than previously considered parameters such as the Wiener index, and other commonly used topological indices (see, for example^3^). In light of this, it will be interesting for future research to see how the conduction of a graph relates, mathematically, to the other indices such as the Wiener index.

Secondly, as Sandak and Conduit^11^ puts it, an engineer wants a model that accurately predicts boiling points of the full range of alkanes. Whilst existing models have been successful in predicting boiling points for only a sub-class of alkanes, our model predicts boiling points for the full range of alkanes. As seen above, our model for the considered data sets successfully predicted boiling points with CoD values, $R^2=0.7516, 0.7898$ and 0.6488. Whilst these CoD values are considered satisfactory, one way of improving accuracy of the model is to include more special graphs that generate data for alkanes of order *n* (see, for instance Table 6).

## References

[1] Burch KJ, Wakefield DK, Whitehead EG (2003). Boiling Point Models of Alkanes. MATCDY.

[2] Dearden JC (2003). Quantitative structure property relationships for prediction of boiling point, vapor pressure, and melting point. Environ. Toxicol. Chem..

[3] Nikolić S, Trinajstić N, Mihalić Z (1995). The Wiener Index: Development and applications. Croat. Chem. Acta.

[4] Randić M, Mihalić Z, Nikolić S, Trinajstić N (1993). Croat. Chem. Soc..

[5] Wiener H (1947). Structural determination of paraffin boiling points. J. Am. Chem. Soc..

[6] Gutman I (1994). Selected properties of the Schultz molecular topological index. J. Chem. Inf Comput. Sci. bf.

[7] Dankelmann, P. & Mukwembi, S. The distance concept and distance in graphs. In *Distance in Molecular Graphs—Theory* (eds. Gutman, I. & Furtula, B.) 3–48 (Univ. Kragujevac, Kragujevac, 2012)

[8] Espinosa G, Yaffe D, Cohen Y, Arenas A, Giralt F (2000). Neural network based quantitative structural property relations (QSPRs) for predicting boiling points of aliphatic hydrocarbons. J. Chem. Inf. Comput. Sci..

[9] Jolliffe IT, Jorge C (2016). Principal component analysis: A review and recent developments. Philos. Trans. R. Soc. A Math. Phys. Eng. Sci..

[10] Montazer E, Salami E, Yarmand H, Kazi SN, Badarudin A (2017). The RSM approach to develop a new correlation for density of metal-oxide aqueous nanofluids. IOP Conf. Ser. Mater. Sci. Eng..

[11] Santaka P, Conduit G (2019). Predicting physical properties of alkanes with neural networks. Fluid Phase Equilib..

